# Naturally Inspired Coumarin Derivatives in Alzheimer’s Disease Drug Discovery: Latest Advances and Current Challenges

**DOI:** 10.3390/molecules29153514

**Published:** 2024-07-26

**Authors:** Rebecca Orioli, Federica Belluti, Silvia Gobbi, Angela Rampa, Alessandra Bisi

**Affiliations:** Department of Pharmacy and Biotechnology, Alma Mater Studiorum-University of Bologna, Via Belmeloro 6, I-40126 Bologna, Italy; rebecca.orioli3@unibo.it (R.O.); federica.belluti@unibo.it (F.B.); silvia.gobbi@unibo.it (S.G.)

**Keywords:** coumarin, Alzheimer’s disease, polypharmacology, cholinesterases

## Abstract

The main feature of neurodegenerative diseases, including Alzheimer’s disease, is the network of complex and not fully recognized neuronal pathways and targets involved in their onset and progression. The therapeutic treatment, at present mainly symptomatic, could benefit from a polypharmacological approach based on the development of a single molecular entity designed to simultaneously modulate different validated biological targets. This strategy is principally based on molecular hybridization, obtained by linking or merging different chemical moieties acting with synergistic and/or complementary mechanisms. The coumarin core, widely found in nature, endowed with a recognized broad spectrum of pharmacological activities, large synthetic accessibility and favourable pharmacokinetic properties, appears as a valuable, privileged scaffold to be properly modified in order to obtain compounds able to engage different selected targets. The scientific literature has long been interested in the multifaceted profiles of coumarin derivatives, and in this review, a survey of the most important results of the last four years, on both natural and synthetic coumarin-based compounds, regarding the development of anti-Alzheimer’s compounds is reported.

## 1. Introduction

**Figure 1:**
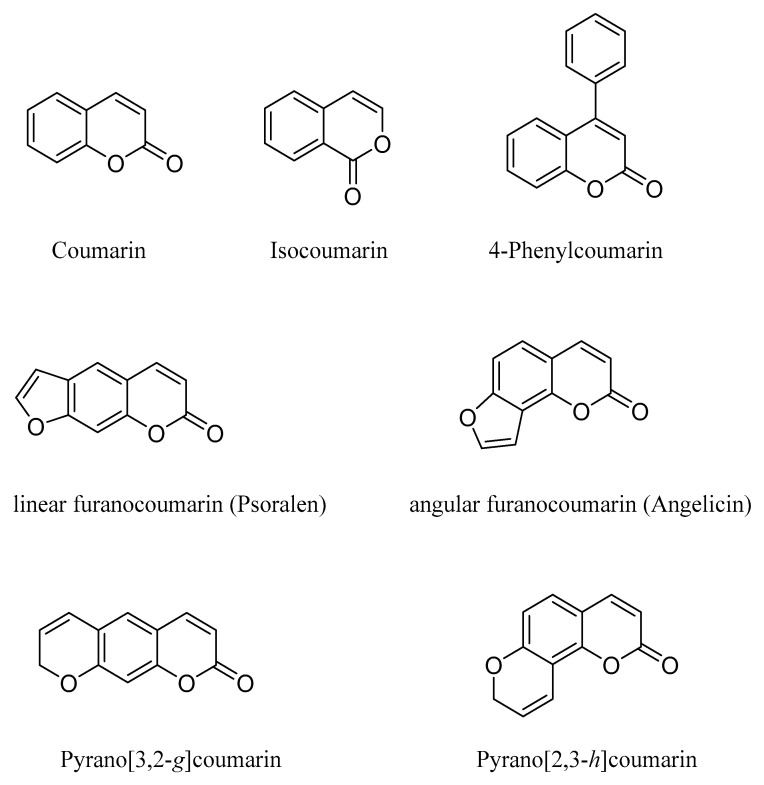
The main classes of coumarins.

Coumarins are oxygen-containing heterocyclic compounds strictly related to flavonoids, isolated for the first time in 1820 from melilot or sweet clover flowers and tonka bean (“coumarou” in French) and found in various species of plants. Presently, at least 1300 different coumarins have been identified from natural sources, mainly from green plants, but also from fungi and microorganisms [1,2]. From a chemical point of view, the coumarin core is a 2*H*-chromen-2-one or benzopyran-2-one and can be considered an isomer of the benzopyran-4-one (chromone) found in the structure of flavones. As for flavonoids, naturally occurring coumarins can be classified on the basis of their chemical diversity as simple coumarins, isocoumarins, furanocoumarins, 4-phenylcoumarins and pyranocoumarins (Figure 1).

This core structure, endowed with low molecular weight, high solubility and bioavailability and low toxicity, has always appeared as a favourable, privileged structure worthy of further development. Their broad spectrum of pharmacological activities makes coumarins a valuable platform in medicinal chemistry from which to search for new drug candidates [3,4]. Indeed, they possess several promising biological effects, including anti-inflammatory, neuroprotective, antioxidant, antidiabetic, anticoagulant, antimicrobial, and antiproliferative effects [5]. Moreover, their luminescent properties allowed researchers to widely exploit this scaffold as a fluorescent probe. Over the years, mainly due to the quite simple synthetic procedure and the reactivity of the benzene and pyrone rings, a huge number of synthetic derivatives, endowed with increasingly targeted activities, have joined the known natural coumarins. Consequently, the literature reports several interesting reviews dealing with the multifaceted profiles of both natural and synthetic coumarins in many different pathological conditions, including neurodegenerative diseases (NDs), particularly Alzheimer’s disease (AD) [6,7,8].

AD is the most prevalent age-associated ND, mainly characterized by memory loss, progressive cognitive impairment, movement dysfunction, depression and alterations in personality. The neuronal loss, predominantly from the cholinergic system, represents the main hallmark of the disorder, together with the formation of extracellular senile plaques, due to the deposit of amyloid-β peptide (Aβ), and intracellular neurofibrillary tangles (NFTs), generated by the aggregation of hyperphosphorylated tau protein [9]. The cholinergic dysfunction in AD is characterized by a huge reduction in the neurotransmitter acetylcholine (ACh), and its nicotinic and muscarinic receptors, and by increased ACh-esterase (AChE) and butyrylcholine esterase (BChE) activities [10]. Aβ peptide aggregates derive from the amyloid precursor protein (APP), that under pathological conditions is processed by β-secretase (β-site amyloid cleaving enzyme 1, BACE-1), releasing the soluble peptide APP-β and a 99-residue-C-terminal fragment. The latter is further cleaved by the γ-secretase enzyme, leading to the formation of 40- and 42-aminoacid-long peptides (Aβ1-40 and Aβ1-42, respectively), Aβ1-42 being the main component of Aβ senile plaques (amyloidogenic pathway) [11]. On the other hand, NFTs originate from the hyperphosphorylation of microtubule-associated tau protein, and a number of serine-threonine kinases are involved in this pathological pathway. Glycogen synthase kinase-3β (GSK-3β), overexpressed in AD patients’ brains, appears to be crucially involved in this process [12]. The complex networked biochemical alterations involved in neurodegeneration also include oxidative stress (OS), induced by an imbalance between the formation of reactive oxygen species (ROS) and the antioxidant defence mechanisms [13]. Notably, due to its high energy requirements and the relevant presence of peroxidable lipids, the brain is highly susceptible to OS, mainly during ageing. Neuroinflammation is also closely related to the progression of AD and is mainly sustained by glial cells (microglia and astrocytes), accounting for the innate immune system of the central nervous system (CNS), activated by an increase in OS. Glial activation triggers a cascade of events, among which the induction of pro-inflammatory enzymes, such as cyclooxygenase 2 (COX-2), and inducible nitric oxide synthase (iNOS) are included [14].

**Figure 2:**
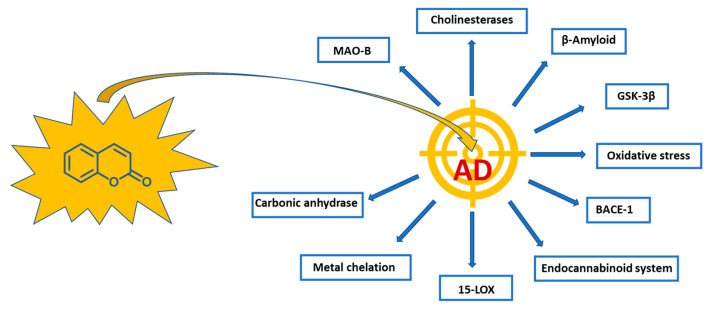
Targets involved in the pathogenesis of AD engaged by coumarin-based compounds.

From this general picture, it is clear that AD has an extremely complex pathology, involving many pharmacological targets to be properly modulated (Figure 2). Despite the recent approval of anti-amyloid monoclonal antibodies, able to improve mild cognitive impairment [15], the availability of small molecules endowed with structural versatility and synthetic accessibility, to be appropriately modified to engage different potential targets, still appears to be of pivotal importance for fighting the multifaceted AD. Indeed, the coumarin nucleus showed the ability to affect various pathways and targets involved in the pathology, including the abovementioned AChE and BChE, BACE-1 and COX-2, but also lipoxygenase (LOX), cannabinoid receptors (CBRs) and fatty acid amide hydrolase enzyme (FAAH), GABA receptors and monoaminoxidases (MAOs), which are similarly considered to be involved in the AD pathogenic mechanisms [16,17]. MAO enzymes are responsible for the oxidative deamination of several amines, among which neurotransmitters such as serotonin and dopamine are included. In particular, the MAO-B isoform, primarily localized in the brain, can be considered one of the most exploited targets in AD [18] since its activity increases with ageing and correlates with AD progression [19,20]. The precise mechanism involved in its neurotoxicity is still largely unknown but is probably related to the formation of toxic byproducts, namely aldehydes, ammonia and H_2_O_2_ during the catalysed biochemical reactions. A huge number of papers have been published on this topic, most of which have recently been reviewed [21,22,23].

This review will survey the most significant results of the last four years regarding both natural and synthetic coumarin derivatives.

## 2. Natural Coumarin Derivatives in AD

**Figure 3:**
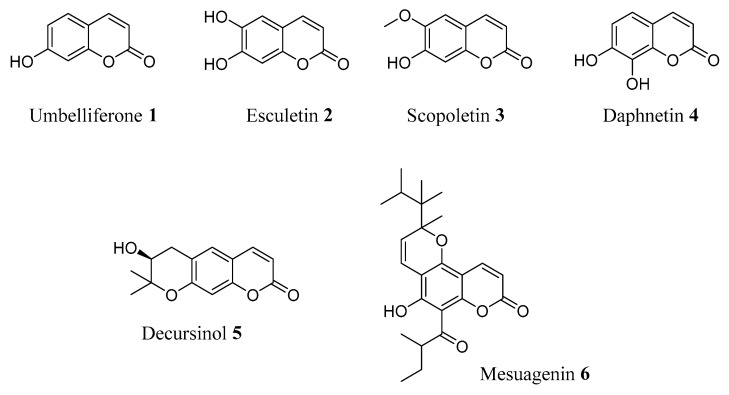
Natural coumarins endowed with anti-AD properties.

Even if the actual mechanism of action of natural compounds is poorly understood, especially in the context of a complex disease such as AD, the interest in naturally occurring coumarins remains very high, and the search for previously unexplored natural products bearing the coumarin core still intrigues and involves many research groups. A number of coumarin-based natural products have indeed been reported as endowed with anti-AD activity, namely umbelliferone (**1**), esculetin (**2**), scopoletin (**3**), daphnetin (**4**), decursinol (**5**) and mesuagenin (**6**) [24,25] (Figure 3).

**Figure 4:**
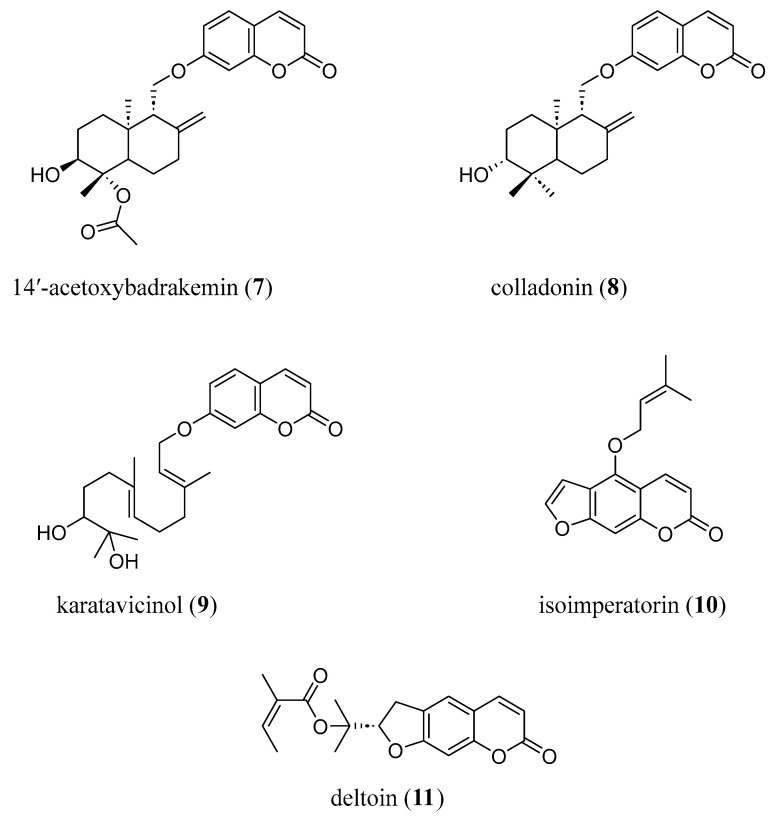
Natural coumarins as dual BChE/AChE inhibitors.

In a recent paper, Orhan et al. evaluated seventeen natural coumarin derivatives (badrakemin, 14′-acetoxybadrakemin, badrakemone, 14′-acetoxybadrakemone, colladonin, colladonin acetate, 14′-acetoxycolladonin, karatavicinol, deltoin, smyrnioridin, marmesin, osthol, oxypeucedanin, oxypeucedanin hydrate, isoimperatorin, scopoletin, and umbelliprenin) against both AChE and BChE and found a selective inhibitory activity against BChE, with 14′-acetoxybadrakemin (**7**), colladonin (**8**), karatavicinol (**9**) and isoimperatorin (**10**) (Figure 4) showing the best inhibitory activity and an increased potency with respect to the reference compound galanthamine. These compounds, together with deltoin (**11**) (Figure 4), also active towards AChE, were then selected to evaluate their binding conformations within the active sites of both ChEs via molecular docking studies. The results agreed with the experimental data: in *h*AChE, the compounds proved to follow the shape of the gorge surface but were unable to establish the appropriate interactions to provide compound/enzyme stabilization. On the contrary, in the larger *h*BChE active gorge, the compounds adopted a more relaxed conformation, forming π-π stacking contacts and H-bonding with oxyanion hole and peripheral anionic site (PAS) residues, consistently with their inhibitory potency against this enzyme [26].

**Figure 5:**
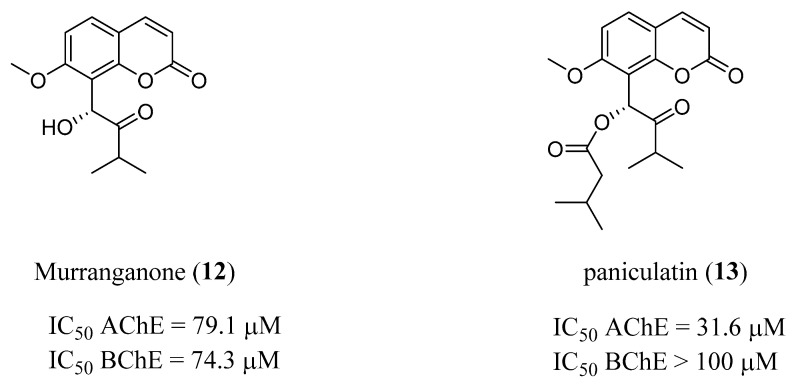
Natural coumarins from *Murraya paniculata* (Orange Jasmin).

A similar approach was applied by Khalid et al. in a very recent paper [27] where three natural coumarin derivatives (2′-*O*-ethylmurrangatin, murranganone (**12**) and paniculatin (**13**), Figure 5), previously isolated by the same group from the leaves of *Murraya paniculata*, were tested in vitro against AChE and BChE. The IC_50_ values indicated **13** as the most active compound against AChE (IC_50_ = 31.6 μM), followed by **12** (IC_50_ = 79.1 µM), that in turn proved to be the most active compound against BChE (IC_50_ = 74.3 μM) (Figure 5). 2′-*O*-ethylmurrangatin appears to be inactive in both enzymes. Classical inhibition kinetics were also investigated, and from the obtained Ki values, **12** appeared as a mixed-type inhibitor of both enzymes, slightly more potent against AChE, whereas **13** behaved as a non-competitive AChE inhibitor and a BChE mixed-type inhibitor. A molecular docking study was applied to evaluate the structural features responsible for the molecular recognition pattern involved in ChEs inhibition and it was observed that the binding to the enzymes was deeply affected by the H-bonds formed by the keto group and the hydroxyl functionalities at different positions on the main structure, while the isopropyl group at the C-3′ position was involved in hydrophobic interactions. MD simulation studies confirm that the stability measured for the complex formed by the most potent compound, **13,** was comparable to the standard drug Tacrine. Moreover, these compounds also showed optimal predicted physicochemical properties, namely oral bioavailability and gastro-intestinal and brain permeation, and appeared worthy of further development to improve binding and potency.

Among natural coumarins, Esculetin (**2**, Figure 3), the principal bioactive compound of *Fraxinus rhynchophylla Hance*, is being increasingly studied due to its multifaceted biological profile. Structurally, it is a simple 6,7 dihydroxy coumarin found in many different plant species, such as *Citrus limon* (L.) *Osbeck* (Rutaceae), *Euphorbia lathyris* L. (Euphorbiaceae) and *Aesculus hippocastanum* L. (Sapindaceae) *Artemisia capillaris* var. *acaulis Pamp.* (Compositae), commonly used as herbal medicines in Asian countries [28,29]. Multiple in vitro and in vivo studies corroborated the broad spectrum of biological activities reported for Esculetin, particularly those related to antioxidant, anti-inflammatory and antiapoptotic mechanisms. Regarding its possible role in AD, its potential has recently been reviewed by Zhang et al. [30], and a promising multifaceted therapeutic effect emerged in many different studies. Esculetin showed well-balanced in vitro inhibition of AChE, BChE and BACE1 enzymes. In a different experiment, it proved to significantly impact oxidative stress, inflammatory mediators’ secretion and cholinergic dysregulation in the hippocampus region of rats with Streptozotocin (STZ)-induced AD. Moreover, the expression level of the nuclear factor erythroid 2-related factor 2 (Nrf2), a well-known regulator of cellular resistance to oxidants [31], appeared upregulated, whereas the pathologically increased levels of NF-кB were inhibited, leading to a mitigation of the STZ-induced neurotoxicity.

**Figure 6:**
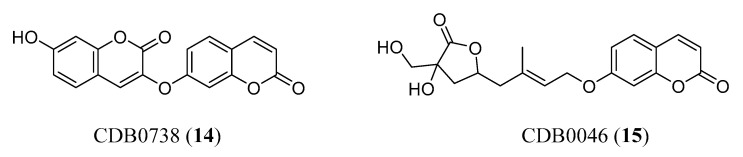
Natural coumarins emerged from a virtual screening on MAO-B and AChE.

In a recent paper, Boulaamane et al. reported the construction of a chemical library, including all available naturally occurring coumarins found in the literature, in order to obtain a tool that will enable scientists to identify potential lead compounds. PubMed was employed as a search engine to find the available literature on coumarin-containing compounds identified from natural sources. Aiming at identifying dual-acting natural coumarins, a multistage virtual screening against MAO-B and AChE (two relevant enzymes involved in AD) was then applied by combining QSAR modelling, molecular docking and ADMET prediction. Indeed, MAO-B can be considered a significant target in NDs, being involved in the formation of neurotoxic free radicals and reactive oxygen species that induce neuroinflammation and apoptosis in neuronal cells [32,33]. From this study, ten coumarin derivatives emerged, potentially acting as dual-target compounds against MAO-B and AChE. Two of them, CDB0738 and CDB0046 (**14** and **15**, Figure 6), were selected from the molecular docking study, showing the ability to interact with critical amino acids of both enzymes and a suitable ADMET profile. Moreover, analysis of molecular interactions for AChE revealed critical interactions with central anionic site (CAS) and PAS residues, similar to the well-known inhibitor donepezil. Obviously, the proposed candidates emerging by using this tool will need to be carefully evaluated in experimental in vitro and in vivo studies to confirm their effectiveness [34].

Maybe due to the lack of “disease-modifying” small molecules for a multifactorial and devastating disease such as AD, the interest in natural compounds endowed with multiple mechanisms of action appears to be continuously increasing. Some natural coumarins have been tested in mouse models of AD aimed at defining the pharmacological basis of their action [35]. Daphnetin (**4**, Figure 3), a natural coumarin derivative endowed with anti-inflammatory and antioxidant activities, has been evaluated in an amyloid precursor protein (APP)/presenilin 1 (PS1) double-transgenic mouse model of AD (APP/PS1 mice), showing the ability to improve memory and spatial learning. This compound proved to downregulate the enzymes responsible for APP processing (BACE-1, nicastrin and PEN2), leading to a marked reduction of Aβ-40 and Aβ-42 levels in the cerebral cortex. Moreover, **4** suppressed astrocyte activation and reduced serum levels of proinflammatory cytokines, such as interleukin-1β (IL-1β), interleukin-6 (IL-6) and tumour necrosis factor-α (TNF-α), probably through the inhibition of the STAT3/GFAP pathway. Daphnetin also proved to reduce the expression of Glial Fibrillary Acidic Protein (GFAP), an essential cellular protein, whose upregulation is a marker for reactive gliosis, improving cognitive deficits and proving that astrocytes may be one of its main targets in APP/PS1 mice. Considering that astrocyte reactivity and Aβ deposition are early features of AD, **4** can be considered a promising candidate for both AD prevention and treatment.

**Figure 7:**
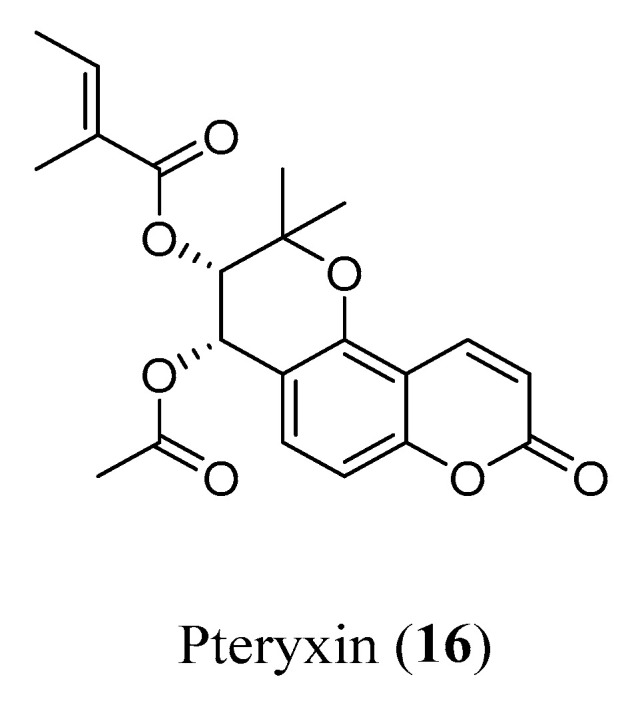
Structure of pteryxin.

To investigate the therapeutic potential of scopoletin (**3**, Figure 3) and pteryxin (**16**, Figure 7) for AD treatment, Baykal et al. tested the compounds in a 12-month-old 5xFAD mouse model of AD and evaluated their effects on spatial learning and memory by way of the Morris water maze test [25]. They also studied the proteome alterations in the brain cortex, cerebellum and hippocampus by exploiting the label-free nanoLC-MS/MS-based protein expression analysis, and modifications in the Aβ load were measured via immunohistochemistry (IHC). Scopoletin (**3**) is a simple, naturally occurring coumarin, endowed with many interesting properties for combatting AD: it is an inhibitor of AChE and a neuroprotective compound, able to reduce neurodegeneration via the activation of anti-oxidant enzymes [36], and showed promising anti-oxidant potential by protecting neuronal living cells against Aβ 25–35-induced cytotoxicity [37]. Pteryxin is a less studied dihydropyranocoumarin that proved to be a better BChE inhibitor than galantamine (IC_50_ = 12.96 and IC_50_ = 22.16 mg/mL, respectively) [38]. These data are supported by molecular docking experiments, showing the different possible binding modes of **16** within the binding pocket of BChE. The 12-month-old 5xFAD mouse model was selected considering that the altered gene expression and its associated pathways in this model were similar to human AD. This study assessed a cognitive improvement after 7 days of intraperitoneal injection administration (16 mg/kg), in particular with **16**. In detail, even though no substantial changes were noticed in the Aβ plaque burden, proteomic analysis of the mice’s cortices showed that proteins involved in Aβ pathology, such as AβPP, GFAP and ApoE, were restored by treatment with **16**. Moreover, **16** increased the expression levels of proteins involved in cognition and synaptic plasticity, suggesting the ability to reverse or halt the progression of late stages of AD [25].

## 3. Synthetic Coumarin Derivatives in AD

### 3.1. Multipotent Coumarin-Based Derivatives Mainly Focused on ChEs Inhibition

The exploitation of natural products as sources of synthetic or semisynthetic derivatives is invariably considered extremely relevant due to the intrinsic ability of natural compounds to interact with a wide range of different proteins, which enables them to bind with diverse cellular targets. This approach has been extensively investigated in medicinal chemistry, i.e., by designing pseudo-natural products, combining different fragments from natural sources with the aim to explore the chemical space and find new possible biological targets [39] or by purposely combining scaffolds with synergistic biological effects to obtain chimeric molecules with improved potency for the selected target(s) [40]. The latter strategy is widely exploited to obtain hybrid compounds, also referred to as multitarget-directed ligands (MTDLs), with an improved biological profile towards specific pathogenic pathways, in particular when the core structure is synthetically accessible.

**Figure 8:**
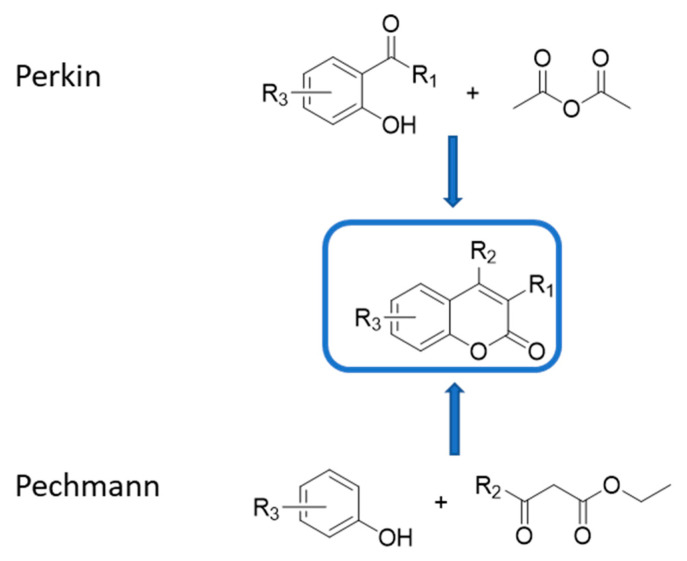
Most common synthetic approach to coumarin derivatives.

Coumarins can be obtained by applying many versatile synthetic methods, among which the Perkin and Pechmann reactions are the best known (Figure 8) [41].

The Perkin method is commonly performed by reacting properly substituted salicylaldehydes with carboxylic acid anhydrides, while Pechmann found that coumarin derivatives can be synthesized by condensing β-ketoesters with phenols in the presence of concentrated sulfuric acid. Both methods have been extensively modified over the years, allowing them to overcome their limitations and obtain a huge number of variously modified derivatives. Therefore, the hybridization strategy applied to coumarins appears particularly profitable, and due to the ability of coumarins to engage ChEs, a number of studies have been performed which aimed at designing potent inhibitors of these enzymes, possibly endowed with other convenient properties.

**Figure 9:**
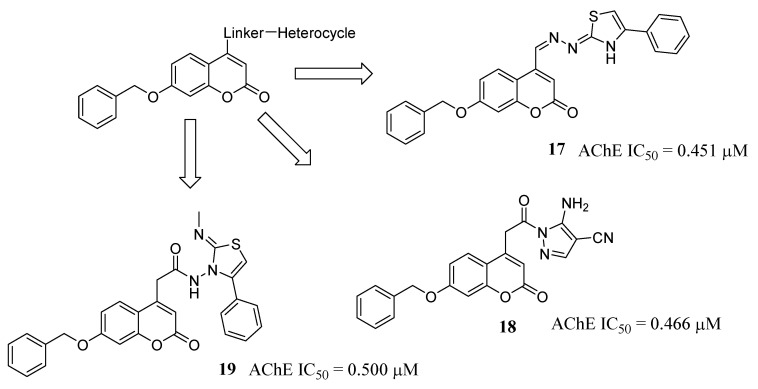
Design of hybrids based on 7-benzyloxycoumarin and various heterocycles, connected by nitrogen-bearing linkers.

A ligand-based approach, connecting different privileged structures endowed with different neurodegenerative effects, was applied by the group led by El-Zoheiry [42], who synthesized twenty novel 7-benzyloxycoumarin-based compounds bearing a variety of bioactive chemical fragments, selected based on their presence in many reported AChE inhibitors (AChEIs). In particular, different heterocycles were connected to position 4 of the 7-benzyloxycoumarin core through appropriate linkers, selected with the aim to correctly span the distance between PAS and CAS and to stabilize the molecule inside the gorge of AChE. Among these, nitrogen-bearing spacers, such as hydrazonomethyl, acetamide and acetohydrazide, recurrent in potent reported AChEIs, were preferred. Some compounds showed promising AChE inhibitory activity, even better than donepezil, used as a reference compound. Kinetic studies indicated the most active derivative, **17** (Figure 9), as a mixed-type inhibitor, able to bind both PAS and CAS of AChE, and molecular docking studies at the active site of recombinant human AChE corroborated the experimental data. The most promising compounds, **17**, **18** and **19** (Figure 9), were evaluated in the scopolamine-induced impairment in vivo model, showing a relevant memory improvement in tested mice.

**Figure 10:**
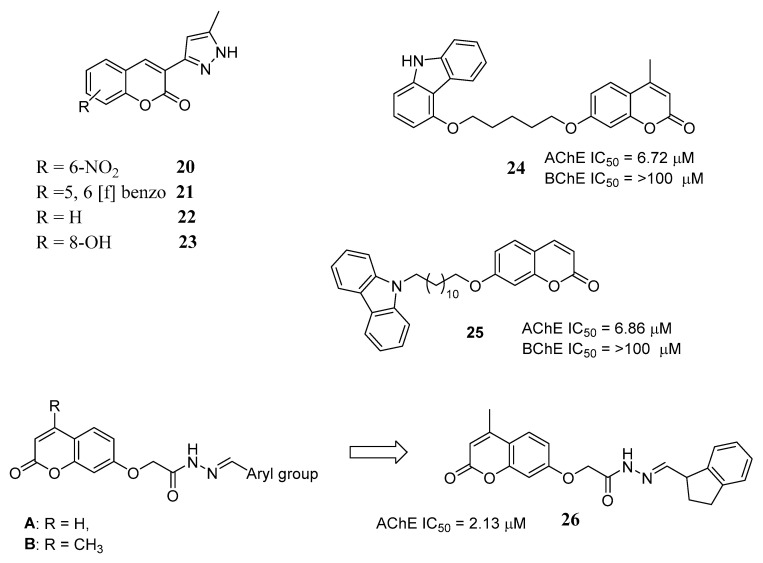
Hybrids coumarin-pyrazole, coumarin-carbazole and coumarin-aromatic/heterocycles.

The pyrazole pharmacophore, found in compound **18** and endowed, among other interesting properties, with the ability to inhibit ChEs and Aβ amyloid aggregation [43], was also exploited by Benazzouz-Touami et al., who linked this structure to the coumarin scaffold in an effort to obtain potent AChE and BChE inhibitors [44]. In this series, in vitro studies showed that compounds **20** and **21** (Figure 10) were the most effective as AChE inhibitors, while **22** and **23** (Figure 10) were more potent as BChE inhibitors, with an IC_50_ value comparable to galantamine, used as a reference compound. These data were supported by molecular docking studies that indicated compound **20**’s ability to occupy the PAS of AChE. Interestingly, all pyrazole-coumarin hybrids showed significant antioxidant activities.

In a similar approach, Shi et al., as a follow-up to a previous study [45], designed potential dual-binding AChE inhibitors by bridging the coumarin moiety, as a ligand for the PAS, with carbazole, a natural-derived privileged structure also reported in some AChE inhibitors, able to target the CAS and endowed with Aβ-antiaggregating and antioxidant properties [46]. The new hybrids showed an in vitro moderate inhibitory effect against AChE, revealing the pivotal role of the spacer length. The most interesting compound of the series (**24**, Figure 10) showed good potency for AChE with good selectivity over BChE. Docking studies confirmed that this molecule could interact with both CAS and PAS of AChE, paving the way for further studies.

Continuing on this path, Tharamak et al. synthesized [47] a new series of hybrids bearing the carbazole and the coumarin moieties, linked by a hydrophobic long hydrocarbon chain or hydrophilic piperazine scaffold, to discover new potent dual-binding AChEIs. The most effective AChEI was **25** (Figure 10), bearing a long 12-methylene chain and showing high selectivity over BChE. Kinetic studies displayed its mixed-type inhibition, also confirmed by molecular docking studies. Notably, **25** did not show toxicity when tested on HepG2 and Vero cells.

A related strategy was applied by Kamel et al., who designed a series of hybrid derivatives obtained by connecting coumarin and 4-methylcoumarin with aromatic and heteroaromatic moieties through a 7-oxymethylene acetohydrazide linker (general structures **A** and **B**, Figure 10), which should stabilize the molecule inside the AChE gorge thanks to the contribution of its nitrogen atoms and carbonyl group [24]. The newly synthesized compounds were evaluated as AChEIs and antioxidant agents, and donepezil and ascorbic acid were used as reference drugs, respectively. The results indicated compound **26** (Figure 10) as the most promising, endowed with a significant AChE inhibitory potency and a good 2,2-diphenyl-1-picrylhydrazyl (DPPH) scavenging activity. When tested in vivo, this derivative showed irrelevant changes in the blood biochemical profile with respect to untreated rats. In particular, the hepatic enzyme levels and the total urea appeared unmodified, and no histopathological damage was observed in the examined tissues, namely the liver, kidney, heart and brain. Furthermore, the T-maze test revealed a significant improvement in cognitive function, comparable to donepezil. In addition, the pharmacokinetic properties of **26** appeared very favourable, with a good oral bioavailability, a high human intestinal absorption, and a safe toxicity profile.

**Figure 11:**
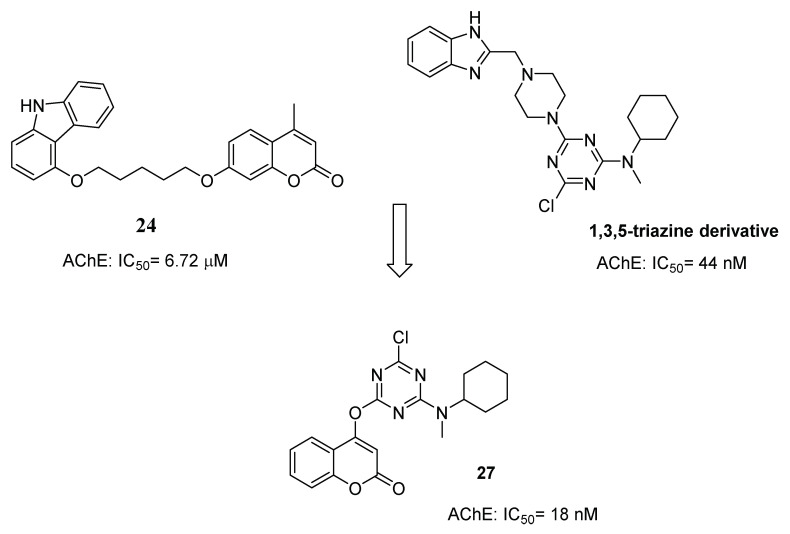
Design strategy for coumarin-1,3,5-triazine derivatives.

After having developed coumarin-carbazole hybrids (**24**), Shi’s group turned its attention towards the 1,3,5-triazine scaffold, a six-membered aromatic ring containing three nitrogen atoms and widely used to design hybrid molecules endowed with antioxidant, ChEs and BACE-1 inhibition properties [48] to obtain new potential AChEIs [49]. The coumarin scaffold was then linked to the 1,3,5 triazine core, and among the obtained derivatives, **27** (Figure 11) emerged as the most potent, showing an inhibitory activity comparable to donepezil as a reference compound. Enzyme kinetic studies, molecular docking, molecular dynamics simulation studies and binding free energy calculation experiments demonstrated that **27** could stably interact with both the CAS and PAS of AChE [50].

**Figure 12:**
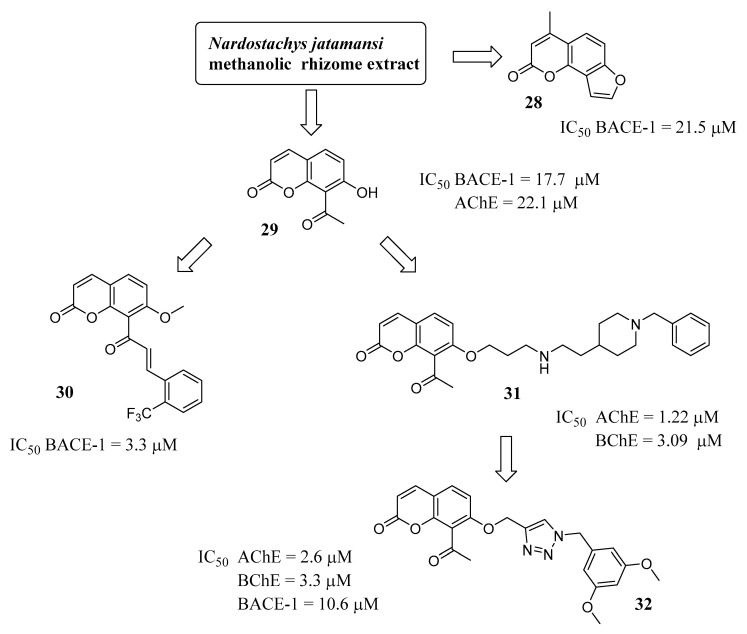
Synthetic derivatives inspired by natural compounds.

The hybridization of coumarins obtained from natural sources with the *N*-benzylpiperidine function of donepezil has been explored in an interesting paper by Sharma et al. [51] that started with a phytochemical investigation of *Nardostachys jatamansi*, whose rhizomes were sequentially treated with different solvents and the extracts were tested on AChE, BChE and BACE-1 enzymes. The methanolic extract gave weak but promising results and was then purified through a chromatographic column to separate the components: five secondary metabolites, among which 4-methylangelicin (**28**) and 8-acetyl-7-hydroxycoumarin (**29**) (Figure 12) showed inhibitory potency in the micromolar range on the selected targets. Aiming at improving activity, a lead optimization strategy was applied starting from **29**, a well-balanced AChE/BACE-1 inhibitor (IC_50_ = 22 µM and 17 µM, respectively), leading to two different series of compounds by exploiting the acetyl group in the Claisen-Schmidt condensation to obtain chalcones (series 1) or linking side chains to the hydroxyl group (series 2). From these semisynthetic modifications, two compounds emerged, endowed with different activity profiles: the trifluoromethyl substituted chalcone **30** (series 1) and the coumarin-donepezil hybrid **31** (series 2). Compound **30** showed an improved potency for BACE-1 inhibition (IC_50_ = 3.3 µM), while **31** was an effective dual-binding AChE/BChE inhibitor (IC_50s_ = 1.22 and 3.09 µM, respectively). The coumarin-donepezil hybrid (**31**) also proved to have a significant inhibitory effect on Aβ self-aggregation while showing a poor inhibition of BACE-1 (29% at 10 µM). In a follow-up study [52], in order to improve the activity on BACE-1, the same research group further modified the side chain of **31** by introducing a functionalised 1,2,3-triazole moiety that was reported as able to engage the catalytic sites of ChEs and BACE-1, as well as to hamper Aβ self-aggregation. Derivative **32**, the most active of this series, proved to be a good MTDL, showing a balanced potency on the selected enzymes in the low micromolar range (IC_50_ = 2.57, 3.26 and 10.65 µM on AChE, BChE and BACE-1, respectively), and this finding was supported by a molecular dynamic simulation study that indicated strong interactions of **32** with the critical residues of the three enzymes. Moreover, this compound proved to inhibit the self-aggregation of Aβ-monomers and was able to cross the BBB via passive diffusion. The SAR study performed on this series proved that the 3,5-dimethoxybenzyl substituent on the triazole and the acetyl-substituted coumarin core played a pivotal role in activity.

**Figure 13:**
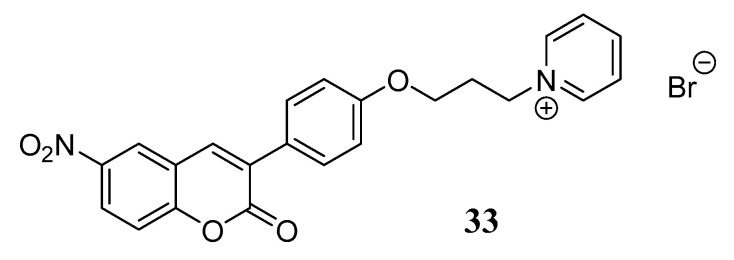
3-arylcoumarin-based pyridinium salt **33**.

It is well known that all marketed AChEIs possess a basic nitrogen atom protonated at physiological pH and involved in the process of binding to the enzyme. Taking this concept into account, the group led by Khoobi has been involved for many years in the development of some coumarin-based derivatives linked to the nitrogen of a pyridine via different spacers as potent AChEIs for AD. The pyridine moiety was selected due to its antioxidant and anti-inflammatory activities [53]. This approach gave rise to pyridinium salts that proved to strongly bind the CAS of AChE by means of its charge and π-stacking interactions. The design of pyridinium salts, albeit suffering from an evident low BBB permeability, has been widely exploited, leading to very potent in vitro ChEs inhibitors. A review published in 2022 [54] reports the most recent advances in this direction and confirms the crucial role of the coumarin scaffold in the conceptualization of such inhibitors. Indeed, in the same year, as a follow-up to previous studies, Khoobi et al. designed novel 3-arylcoumarin-pyridine hybrids, among which compound **33** (Figure 13) emerged as a promising MTDL, showing low-nanomolar potency on both AChE and BChE (IC_50_ = 2 and 24 nM, respectively) and the ability to reduce Aβ-amyloid self- and AChE-induced aggregation. Additionally, a valuable neuroprotective activity against H_2_O_2_-induced cell death in PC12 cells and against amyloid toxic effects in SH-SY5Y cells was also observed.

**Figure 14:**
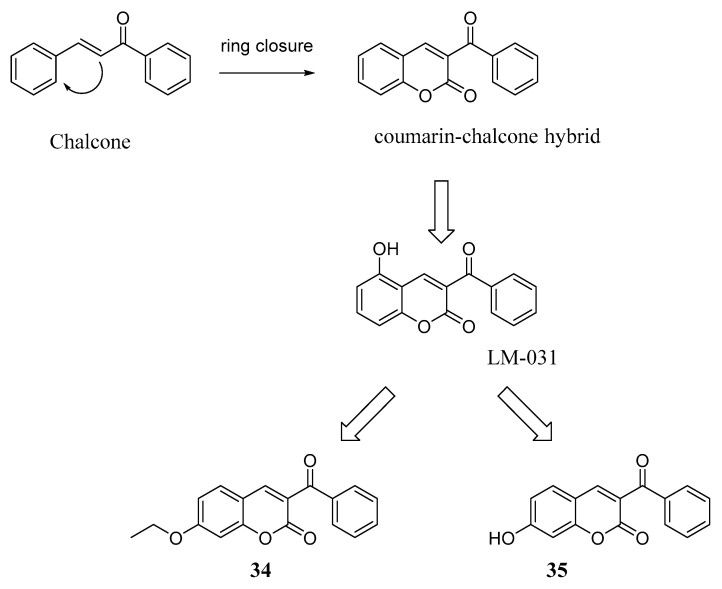
Design of chalcone-coumarin merged derivatives acting as TRKB pathway activators.

Abnormal accumulations of misfolded Aβ and hyperphosphorylated tau proteins represent neuropathological hallmarks of AD and other tauopathies. These abnormal protein deposits are responsible for the onset of oxidative stress and neurodegeneration by means of a number of proposed mechanisms, including a reduction in the Nrf2-mediated transcription of antioxidant genes and downregulation of the cAMP-response-element binding protein 1 (CREB) signalling pathway, also involved in promoting neuroprotection. Notably, the chalcone Licochalcone A, a natural compound found in the root of *Glycyrrhiza inflata*, is able to enhance the Nrf2-mediated defence mechanism against oxidative stress and cell death [55]. The chalcone scaffold is widely found in secondary metabolites of terrestrial plants and represents one of the most studied privileged structures, mainly due to its synthetic accessibility which allows researchers to introduce selected substituents in different positions of both aromatic rings. Moreover, the presence of the α,β-unsaturated carbonyl linker introduces a peculiar reactivity in these compounds that has been extensively exploited in many medicinal chemistry fields. Indeed, a number of biological activities have been ascribed to chalcones, among which potential anti-AChE activity is included [56]. Aiming at counteracting the oxidative stress implicated in AD pathogenesis, Lee et al. reported the design of a series of derivatives obtained by merging the coumarin core, acting as a free radical scavenger, and the chalcone structure of Licochalcone A. Among the obtained compounds, **LM-031** (Figure 14) showed the ability to upregulate CREB, demonstrating promising neuroprotective activity and the potential to reduce oxidative stress, as well as Aβ and tau accumulation [17]. Starting from these premises, the same group designed a small series of **LM-031**-related compounds and investigated their capacity to activate CREB signalling pathways. The obtained results suggested that coumarin-based derivatives **34** and **35** (Figure 14) exerted neuroprotective activity in ΔK280 tau_RD_ SH-SY5Y cells by increasing the CREB signalling pathway through the activation of tropomycin receptor kinase B (TRKB) and by reducing caspase activity [57].

**Figure 15:**
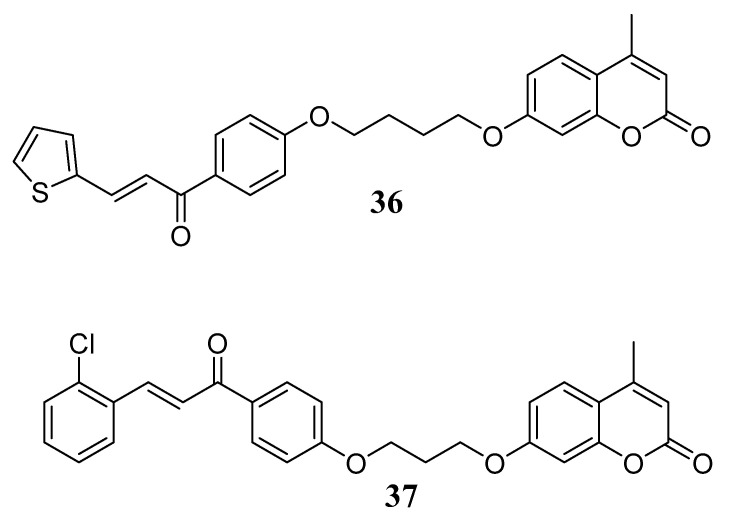
Coumarin-chalcone hybrids as ChEs inhibitors.

Following a similar hybridization strategy, the group led by Jamalis conceived a series of chalcone-coumarin hybrids, introducing in the chalcone core a thiophene ring, considered a promising heterocyclic scaffold due to the presence of the sulphur atom. The obtained thiophene-chalcone system was then connected to the coumarin via aliphatic chains of different lengths, and an in-silico ADMET prediction study was performed in order to select the most appropriate compounds to be synthesized. A small series of hybrids was then prepared and tested to evaluate ChEs inhibitory activities. All compounds showed low-micromolar inhibitory potencies, and **36** (Figure 15) emerged as the most interesting, showing good activity on AChE (IC_50_ = 0.42 µM), with selectivity over BChE and low cytotoxicity. In a follow-up study aimed at increasing the potency and completing the SAR study, the same group broadened the series of coumarin-chalcone hybrids, variously decorating the chalcone moiety. The 2-chloro derivative, **37,** can be considered the most promising hybrid of the series, showing good anti-AChE activity (IC_50_ = 0.201 μM) due to the ability to target the active sites of the enzyme and a low cytotoxicity [58].

**Figure 16:**
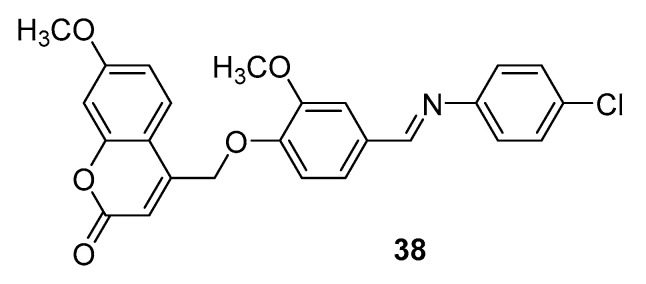
Coumarin-Schiff base hybrid as AChEI.

In their ongoing search for a lead compound, the same research group [59] rationally designed a novel series of hybrid coumarin-Schiff bases as AChEIs. The molecular docking studies showed the ability of the designed compounds to establish significant molecular interactions via hydrogen bonds with key residues of the target enzyme, and the pattern of binding sites and binding energy, together with QSAR descriptors and calculated drug likeness, indicated **38** (Figure 16) as the optimal candidate for AChE inhibition in this set of derivatives. These in silico data correlated with the experimental results, being compound **38** with an IC_50_ value of 0.19 µM, the most potent in the series, ~fivefold more potent than galantamine, used as a reference compound.

### 3.2. Coumarins Acting on Different Selected Targets

**Figure 17:**
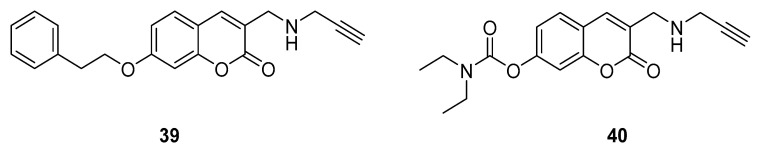
Coumarin-propargylamine derivatives.

As previously stated, the versatility of the coumarin nucleus and its ability to be easily synthesized and substituted allows it to be considered a suitable platform for the design of hybrid small molecules able to interact with multiple targets involved in AD. This multifunctional approach, leading to a plethora of MTDL compounds that have been widely reviewed [60,61], is still particularly attractive and, besides ChEs, involves many other pathways related to AD progression. In 2021, Mzezewa reported the preliminary data for inhibitory activity on ChEs and MAO-B, cytotoxicity and the neuroprotective ability of a series of 3,7-substituted coumarin derivatives. The compounds were designed by introducing to the coumarin scaffold different groups reported as endowed with MAO inhibition (propargylamine), AChE/MAO inhibition (phenethoxy) and AChE/neuroprotection (carbamate) properties. Most compounds showed significant in vitro neuroprotective effects towards MPP^+^-compromised SH-SY5Y neuroblastoma cells and appreciable inhibition and selectivity towards MAO-B, with IC_50_ values ranging between 0.014 and 0.498 µM. Despite the lack of ChEs inhibition ability, the most active derivatives, **39** and **40** (Figure 17), bearing the propargylamine functional group, displayed the best potential as MAO-B inhibitors (IC_50_ = 14 and 101 nM, respectively) and a favourable cytotoxicity profile. Moreover, in silico studies using online prediction tools suggested suitable pharmacokinetic and drug-like properties [32].

**Figure 18:**
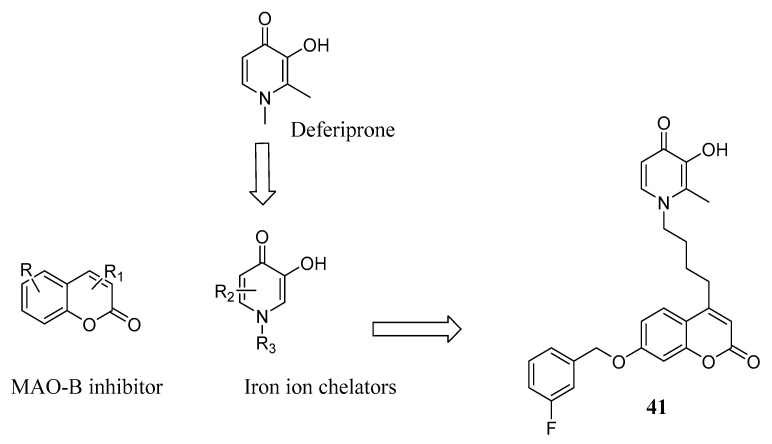
Design of hybrid **41**.

Applying a similar target-based approach, Guo et al. [62] designed a series of hybrid compounds by combining the iron-chelating 3-hydroxypyridin-4(1*H*)-one and the MAO-B inhibitor coumarin pharmacophores, aiming at obtaining MTDL agents (Figure 18). 3-hydroxypyridin-4(1*H*)-one is structurally related to deferiprone, a chelating drug used for treating iron overload in different pathologies, such as thalassemia [63], and can be easily modified, allowing for a wide SAR study. All the new derivatives showed good Fe^2+^-chelating properties and favourable MAO-B inhibitory activity, with compound **41** being the most promising with an IC_50_ = 99.3 nM, very close to the corresponding activity value of the selective MAO-B inhibitor pargyline (IC_50_ = 86.9 nM), used as a reference drug. These data were corroborated by molecular docking analysis, indicating that compound **41** could occupy both the entrance and the substrate cavities of MAO-B. Notably, this derivative also exhibited a relevant antioxidant effect and proved to protect PC12 cells from the damage induced by Aβ. BBB penetration ability was evaluated by applying in silico tools, and **41** appeared to be able to overcome the challenge of brain exposure. These results were confirmed in a behavioural study of mice, where compound **41** significantly improved the scopolamine-induced cognitive impairment.

**Figure 19:**
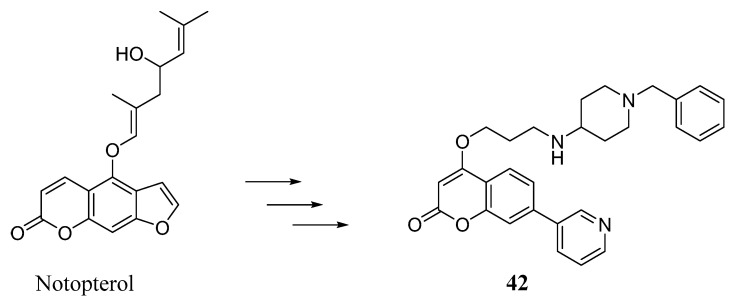
Design strategy for the naturally inspired derivative **42**.

In a different approach, Zhao et al. started from a previous study on notopterol (Figure 19), a furanocoumarin-based natural compound endowed with many biological activities, among which is the ability to inhibit both GSK-3β and BACE-1 enzymes, even if to a different extent. Aiming at driving the activity toward AChE as well, the structure of the lead notopterol was purposely modified by introducing the *N*-benzylpiperidine function found in donepezil and also expected to be useful for increasing BACE-1 inhibitory activity. Moreover, the furan portion of notopterol was removed, with the idea of reducing toxicity, while maintaining the AChE and GSK-3β targeting ability. Compared with notopterol, the 27 new coumarin-donepezil hybrids obtained showed promising AChE inhibition, good selectivity with respect to BChE, a moderate ability to inhibit GSK-3β and an impressive increase in BACE-1 inhibitory activity; all these data were supported by docking studies. The most active compound (**42**) behaved as a competitive AChEI endowed with good BBB permeability, acceptable predicted physicochemical properties and a tolerable cytotoxicity profile, confirmed by in vivo acute toxicity experiments [64].

**Figure 20:**
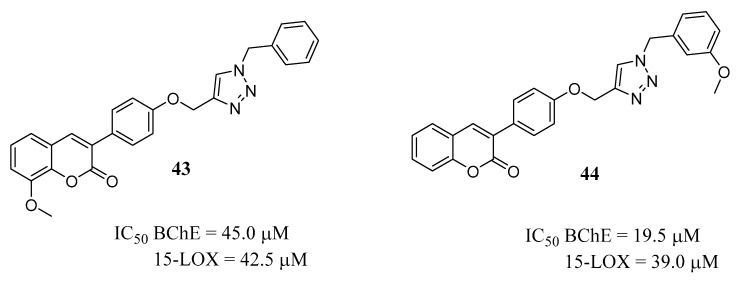
*N*-benzyltriazole derivatives with dual BChE and 15-LOX activity and neuroprotective and anti-aggregating properties.

Continuing their research program on coumarin derivatives as MTLDs for AD treatment, the group led by Khoobi planned to introduce a *N*-benzyl triazole substructure on the 3-arylcoumarin core, aiming at engaging both ChEs and 15-LOX [65], the enzyme responsible for the peroxidation of specific atoms in polyunsaturated fatty acids and found to be involved in ND development, including AD [66,67]. The phenyl group in position 3 of the coumarin core could allow for the strengthening of the hydrophobic interactions in the active site of 15-LOX, and the triazole was introduced due to its recognized ChEs inhibition potential [68]. Both the coumarin core and the benzyl triazole were variously substituted and the new compounds were evaluated on the selected enzymes. In general, all the reported 3-arylcoumarins proved to be selective for BChE inhibition over AChE, and most of them showed significant inhibitory activity on LOX, with an acceptable toxicity profile. The most balanced dual inhibitors (derivatives **43** and **44**, Figure 20) were selected to evaluate their neuroprotective effect against H_2_O_2_-induced P-12 cell death and the anti-amyloid effects on both self- and AChE-induced aggregation. Derivative **43** was the most potent neuroprotective compound and Aβ self-aggregation inhibitor but appeared unable to counteract the enzyme-induced Aβ aggregation, probably due to the low inhibitory activity on AChE.

**Figure 21:**
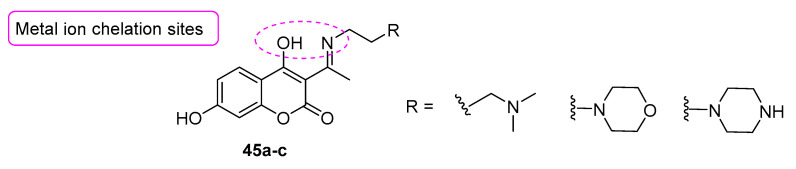
Design of metal-chelating coumarin-based compounds.

A coumarin-based MTDL approach was also followed by Wang et al. [69] who developed a series of 4,7-dihydroxy coumarins carrying a Schiff base in the 3-position, so that the nitrogen and the 4-hydroxy substituent in the coumarin core could act as chelating sites, able to form a stable six-membered ring when coordinating with metal ions (Figure 21). Moreover, tertiary amino groups were inserted both to improve the anti-ChE abilities and to optimize the lipid/water partition coefficient.

The anti-AD activities of **45a-c** were studied both in vitro and in silico. The results showed that the compounds were endowed with potent antioxidant activities, related to the ability of the 7-OH group to stabilize the phenoxy radical, with good inhibition activity on AChE and Aß aggregation and with the ability to selectively chelate Cu^2+^ and Al^3+^, regulating metal homeostasis. Furthermore, ADMET performance prediction data indicated that the compounds were able to cross the BBB.

### 3.3. Coumarins as MTDLs for Emerging Targets

**Figure 22:**
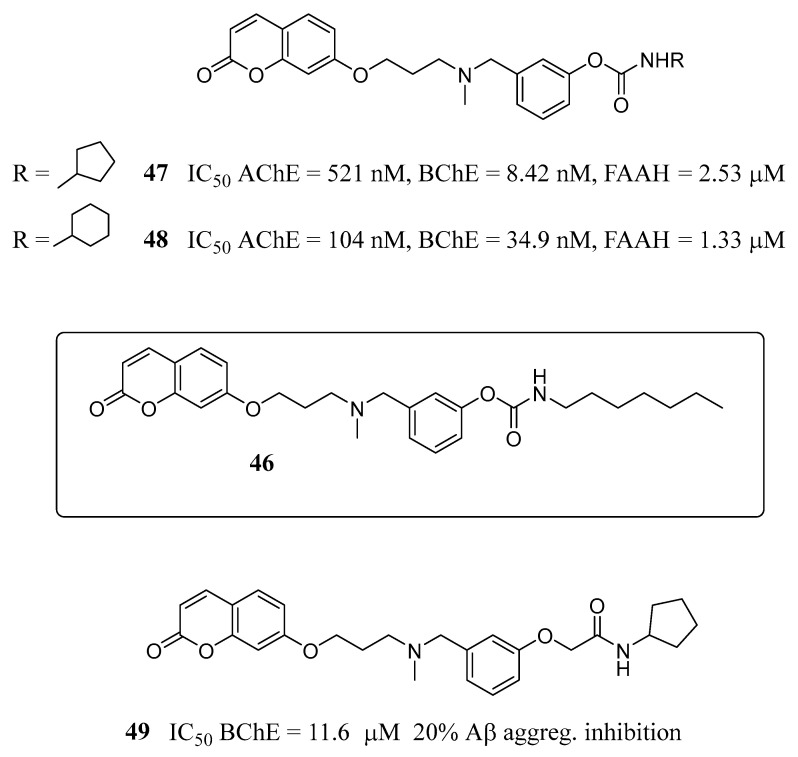
Development of ChEs-FAAH inhibitors.

One of the main challenges in MDTL research for multifactorial disease management is the assessment of the role of new potential targets and their engagement by specific pharmacophores. In recent years, the endocannabinoid system (ECS) appears increasingly involved in neuroprotection, since its activation can suppress microglial activation, thus hampering neurodegenerative processes. The ECS consists of endocannabinoids (eCBs), a relatively small group of endogenous fatty acid derivatives (mainly anandamide and 2-arachidonoylglycerol), acting on two CBRs, CB1 and CB2, and a set of enzymes responsible for their synthesis and breakdown [70]. The enhancement of eCB signalling can be attained by acting on CBRs or, indirectly, by inhibiting FAAH, the enzyme responsible for the inactivation of eCBs. The latter approach appears more promising for the chronic treatment required by AD, thanks to a lower risk of psychotropic side effects related to a prolonged stimulation of receptors, particularly CB1. A simultaneous inhibition of ChEs and FAAH could then represent a valuable therapeutic option to achieve a more effective AD treatment and counteract its progression [71]. In continuing their studies in this field, Montanari et al. [72] reported a series of coumarin-based carbamic and amide derivatives, designed as MTDL compounds acting on ChEs and eCS-related targets, namely FAAH and cannabinoid receptors. Moreover, their ability to reduce Aβ self-aggregation was also assessed. The lead compound, **46** (Figure 22), a coumarin-based derivative previously reported by the same group as a very potent inhibitor of BChE (IC_50_ = 1.86 nM) and a well-balanced AChE and FAAH inhibitor (IC_50_ = 37.4 nM e 28.5 nM, respectively) [73], was modified in order to expand the SAR study and assess further potential activities. In particular, the carbamate moiety was modified by enclosing the heptyl chain in cyclic structures (cyclopentyl, cyclohexyl and naphthalene) to boost the steric interactions with the binding pocket of the selected enzymes, and by replacing the carbamate with the widely exploited amide group, to evaluate the actual role of the carbamate in enzyme inhibition and the feasibility of achieving reversible inhibitors. From the results, the carbamates **47** and **48** emerged as promising inhibitors of ChEs, FAAH and Aβ self-aggregation, endowed with calculated brain-to-blood ratios better than the n-heptyl lead, **46**. Interestingly, the amide derivative, **49**, showed promising BChEs and Aβ self-aggregation inhibitory activities and a potential CB1/CB2 receptor affinity.

**Figure 23:**
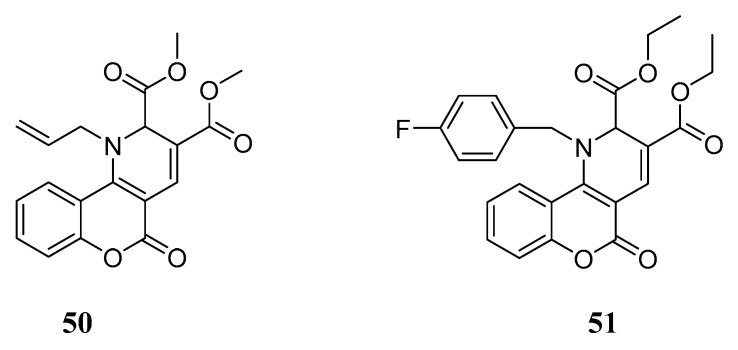
Merged coumarin-dihydropyridines as ChEs/*h*CA inhibitors.

Another very recent target to be exploited in MTDL design is human carbonic anhydrase (*h*CA). This enzyme catalyses the reversible hydration of CO_2_ to bicarbonate and a proton and plays a pivotal role in many physiological functions, among which is pH regulation. Recent studies demonstrated that acetazolamide, the most widely used inhibitor of *h*CA, may reduce caspase activation, cerebral, vascular and glial Aβ accumulation and weaken gliosis, leading to a cognitive improvement in TgSwDI mice [74]. In an attempt to obtain dual ChEs and *h*CA inhibitors, two series of coumarin-dihydropyridine derivatives were designed by Zahedi et al. based on the molecular merging of effective pharmacophores found in the literature [75]. The new compounds (Figure 23) were tested in vitro and in silico on ChEs and two of the different *h*CA isoforms, namely CA I and CA II. In vitro studies proved that some of the title compounds were more potent AChE and/or BChE inhibitors compared to the reference drug tacrine, while most of them inhibited the selected *h*CA isoforms better than acetazolamide, used as the standard compound, with **50** and **51** being the most promising compounds. For these compounds, in silico studies predicted significant interactions with key residues in the active site of the enzymes and encouraging pharmacokinetic properties.

**Figure 24:**
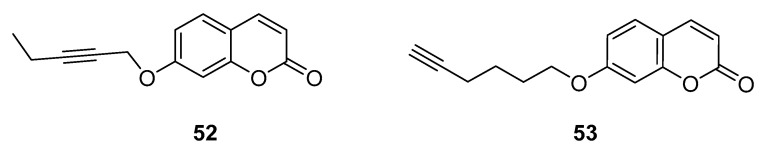
Coumarins acting on MAO-B and CA VII, IX and XII.

In 2023, the Supuran group reported [76] a small library of coumarin derivatives, designed to obtain compounds capable of managing neuroinflammation by the simultaneous inhibition of three enzymes involved in neurodegeneration (CAs, MAOs and ChEs). To achieve this goal, properly selected alkyl chains were introduced at positions 4, 6 and 7 of the coumarin scaffold, and position 7, considered as a privileged position to gain potent *h*MAOs inhibition, was the most explored among the series. Notably, many compounds have been designed to date to engage both *h*MAO and *h*ChE, but in this paper the potential neuroprotective activity of the coumarin scaffold also involving CAs inhibition was investigated for the first time. All the new derivatives proved to effectively and selectively inhibit the *h*CA isoforms of interest (*h*CA VII, IX and XII) over the off-target isoforms *h*CA I and II. The most promising compounds were **52** and **53** (Figure 24), acting as nanomolar and balanced inhibitors of *h*MAO-B and *h*CA isoforms VII, IX and XII. Furthermore, they also displayed weak AChE and BChE inhibitory activities. These compounds proved to permeate the BBB, and further in vitro biological studies using LPS-stimulated rat astrocytes showed their ability to counteract oxidative stress-induced neuroinflammation and to hamper interleukin-6 secretion, thus corroborating the rationale of this multitarget approach.

## 4. Conclusions

The main feature of NDs is their complex aetiology, involving a number of apparently unrelated and still not fully identified neuronal pathways and targets. As a consequence, the “one target, one molecule” paradigm appears unproductive in affecting the progression of these multifactorial disorders, and a more promising strategy involves the development of single molecular entities able to simultaneously modulate different biological targets involved in their onset and progression. This polypharmacological approach is mainly based on the design of hybrid molecules obtained by combining different chemical moieties acting with different mechanisms and exerting complementary and synergistic actions. In this respect, the identification of an appropriate main scaffold, capable of being variously and easily modified, acquires pivotal importance. Coumarins are naturally derived privileged structures in medicinal chemistry and represent suitable scaffolds for the design and development of novel multifunctional compounds for ND treatment. Indeed, besides their favourable physicochemical properties and innate pharmacological activities, their core structure exhibits several positions that could be variously substituted, allowing for a huge variety of different compounds. Therefore, combining coumarins with diverse pharmacophores endowed with selected biological properties may represent a valuable strategy to combat NDs. In this review, the recent literature has been explored and still the coumarin core arises as a valid starting point for hybridization. Focusing on AD, its structure has been combined by means of selected linkers with diverse heterocycles, often leading to promising compounds capable of engaging both the most predictable targets, namely AChE, BChE, MAO-B, GSK-3β and metals, and some unconventional and emerging ones, such as CB receptors and CA and FAAH enzymes, always proving to possess promising activity. The design of coumarin-based hybrid MTDLs is thus a prospective approach to tackle effective treatments for NDs, in particular AD. Nevertheless, additional studies are mandatory to address several issues arising from the polypharmacological strategy, in particular regarding the BBB permeation, the pharmacokinetic properties and the possible off-target adverse effects of the new hybrid compounds.

## References

[1] Borges F., Roleira F., Milhazes N., Santana L., Uriarte E. (2005). Simple Coumarins and Analogues in Medicinal Chemistry: Occurrence, Synthesis and Biological Activity. Curr. Med. Chem..

[2] Hoult J.R.S., Payá M. (1996). Pharmacological and biochemical actions of simple coumarins: Natural products with therapeutic potential. Gen. Pharmacol. Vasc. Syst..

[3] Annunziata F., Pinna C., Dallavalle S., Tamborini L., Pinto A. (2020). An Overview of Coumarin as a Versatile and Readily Accessible Scaffold with Broad-Ranging Biological Activities. Int. J. Mol. Sci..

[4] Yildirim M., Poyraz S., Ersatir M. (2023). Recent advances on biologically active coumarin-based hybrid compounds. Med. Chem. Res..

[5] Garg S.S., Gupta J., Sharma S., Sahu D. (2020). An insight into the therapeutic applications of coumarin compounds and their mechanisms of action. Eur. J. Pharm. Sci..

[6] Pisani L., Catto M., Muncipinto G., Nicolotti O., Carrieri A., Rullo M., Stefanachi A., Leonetti F., Altomare C. (2022). A twenty-year journey exploring coumarin-based derivatives as bioactive molecules. Front. Chem..

[7] Jameel E., Umar T., Kumar J., Hoda N. (2016). Coumarin: A Privileged Scaffold for the Design and Development of Antineurodegenerative Agents. Chem. Biol. Drug Des..

[8] Husain A., Al Balushi K., Akhtar M.J., Khan S.A. (2021). Coumarin linked heterocyclic hybrids: A promising approach to develop multi target drugs for Alzheimer’s disease. J. Mol. Struct..

[9] Holtzman D.M., Morris J.C., Goate A.M. (2011). Alzheimer’s Disease: The Challenge of the Second Century. Sci. Transl. Med..

[10] Hardy J.A., Higgins G.A. (1992). Alzheimer’s Disease: The Amyloid Cascade Hypothesis. Science.

[11] Qiu T., Liu Q., Chen Y., Zhao Y., Li Y. (2015). A β 42 and A β 40: Similarities and differences. J. Pept. Sci..

[12] Hooper C., Killick R., Lovestone S. (2008). The GSK3 hypothesis of Alzheimer’s disease. J. Neurochem..

[13] Cioffi F., Adam R.H.I., Broersen K. (2019). Molecular Mechanisms and Genetics of Oxidative Stress in Alzheimer’s Disease. J. Alzheimer’s Dis..

[14] Mandrekar-Colucci S., Landreth G.E. (2010). Microglia and Inflammation in Alzheimers Disease. CNS Neurol. Disord. Drug Targets.

[15] Wu W., Ji Y., Wang Z., Wu X., Li J., Gu F., Chen Z., Wang Z. (2023). The FDA-approved anti-amyloid-β monoclonal antibodies for the treatment of Alzheimer’s disease: A systematic review and meta-analysis of randomized controlled trials. Eur. J. Med. Res..

[16] Srikrishna D., Godugu C., Dubey P.K. (2018). A Review on Pharmacological Properties of Coumarins. Mini-Rev. Med. Chem..

[17] Lee S.Y., Chiu Y.J., Yang S.M., Chen C.M., Huang C.C., Lee-Chen G.J., Lin W., Chang K.H. (2018). Novel synthetic chalcone-coumarin hybrid for Aβ aggregation reduction, antioxidation, and neuroprotection. CNS Neurosci. Ther..

[18] Manzoor S., Hoda N. (2020). A comprehensive review of monoamine oxidase inhibitors as Anti-Alzheimer’s disease agents: A review. Eur. J. Med. Chem..

[19] Riederer P. (2004). Monoamine Oxidase-B Inhibition in Alzheimer’s Disease. Neurotoxicology.

[20] Cai Z. (2014). Monoamine oxidase inhibitors: Promising therapeutic agents for Alzheimer’s disease (Review). Mol. Med. Rep..

[21] Koyiparambath V.P., Rajappan K.P., Rangarajan T.M., Al-Sehemi A.G., Pannipara M., Bhaskar V., Nair A.S., Sudevan S.T., Kumar S., Mathew B. (2021). Deciphering the detailed structure–activity relationship of coumarins as Monoamine oxidase enzyme inhibitors—An updated review. Chem. Biol. Drug Des..

[22] Zou D., Liu R., Lv Y., Guo J., Zhang C., Xie Y. (2023). Latest advances in dual inhibitors of acetylcholinesterase and monoamine oxidase B against Alzheimer’s disease. J. Enzym. Inhib. Med. Chem..

[23] Özdemir Z., Alagöz M.A., Bahçecioğlu Ö.F., Gök S. (2021). Monoamine Oxidase-B (MAO-B) Inhibitors in the Treatment of Alzheimer’s and Parkinson’s Disease. Curr. Med. Chem..

[24] Kamel N.N., Aly H.F., Fouad G.I., El-Karim S.S.A., Anwar M.M., Syam Y.M., Elseginy S.A., Ahmed K.A., Booles H.F., Shalaby M.B. (2023). Anti-Alzheimer activity of new coumarin-based derivatives targeting acetylcholinesterase inhibition. RSC Adv..

[25] Kiris I., Skalicka-Wozniak K., Basar M.K., Sahin B., Gurel B., Baykal A.T. (2021). Molecular Effects of Pteryxin and Scopoletin in the 5xFAD Alzheimer’s Disease Mouse Model. Curr. Med. Chem..

[26] Orhan I.E., Tosun F., Deniz F.S.S., Eren G., Mıhoğlugil F., Akalgan D., Miski M. (2021). Butyrylcholinesterase-inhibiting natural coumarin molecules as potential leads. Phytochem. Lett..

[27] Khalid A., Khan W., Zia K., Azizuddin, Ahsan W., Alhazmi H.A., Abdalla A.N., Najmi A., Khan A., Bouyahya A. (2023). Natural coumarins from Murraya paniculata as mixed-type inhibitors of cholinesterases: In vitro and in silico investigations. Front. Pharmacol..

[28] Tomohiro N., Yasuko K., Sei-Itsu M. (1983). Inhibitory effect of esculetin on 5-lipoxygenase and leukotriene biosynthesis. Biochim. Biophys. Acta (BBA)—Lipids Lipid Metab..

[29] Kim Y.R., Park B.-K., Kim Y.H., Shim I., Kang I.-C., Lee M.Y. (2018). Antidepressant Effect of *Fraxinus rhynchophylla* Hance Extract in a Mouse Model of Chronic Stress-Induced Depression. BioMed Res. Int..

[30] Zhang L., Xie Q., Li X. (2022). Esculetin: A review of its pharmacology and pharmacokinetics. Phytother. Res..

[31] Ma Q. (2013). Role of Nrf2 in Oxidative Stress and Toxicity. Annu. Rev. Pharmacol. Toxicol..

[32] Mzezewa S.C., Omoruyi S.I., Zondagh L.S., Malan S.F., Ekpo O.E., Joubert J. (2021). Design; synthesis and evaluation of 3,7-substituted coumarin derivatives as multifunctional Alzheimer’s disease agents. J. Enzym. Inhib. Med. Chem..

[33] Blennow K., Mattsson N., Schöll M., Hansson O., Zetterberg H. (2015). Amyloid biomarkers in Alzheimer’s disease. Trends Pharmacol. Sci..

[34] Boulaamane Y., Kandpal P., Chandra A., Britel M.R., Maurady A. (2023). Chemical library design QSAR modeling and molecular dynamics simulations of naturally occurring coumarins as dual inhibitors of MAO-B and AChE. J. Biomol. Struct. Dyn..

[35] Gao P., Wang Z., Lei M., Che J., Zhang S., Zhang T., Hu Y., Shi L., Cui L., Liu J. (2022). Daphnetin ameliorates Aβ pathogenesis via STAT3/GFAP signaling in an APP/PS1 double-transgenic mouse model of Alzheimer’s disease. Pharmacol. Res..

[36] Gay N.H., Suwanjang W., Ruankham W., Songtawee N., Wongchitrat P., Prachayasittikul V., Pra-chayasittikul S., Phopin K. (2020). Butein, isoliquiritigenin, and scopoletin attenuate neurodegeneration via an-tioxidant enzymes and SIRT1/ADAM10 signaling pathway. RSC Adv..

[37] Lin H.-C., Tsai S.-H., Chen C.-S., Chang Y.-C., Lee C.-M., Lai Z.-Y., Lin C.-M. (2008). Structure–activity relationship of coumarin derivatives on xanthine oxidase-inhibiting and free radical-scavenging activities. Biochem. Pharmacol..

[38] Orhan I.E., Senol F.S., Shekfeh S., Skalicka-Wozniak K., Banoglu E. (2017). Pteryxin—A promising butyrylcholinesterase-inhibiting coumarin derivative from Mutellina purpurea. Food Chem. Toxicol..

[39] Grigalunas M., Brakmann S., Waldmann H. (2022). Chemical Evolution of Natural Product Structure. J. Am. Chem. Soc..

[40] Shaveta, Mishra S., Singh P. (2016). Hybrid molecules: The privileged scaffolds for various pharmaceuticals. Eur. J. Med. Chem..

[41] Citarella A., Vittorio S., Dank C., Ielo L. (2024). Syntheses, reactivity, and biological applications of coumarins. Front. Chem..

[42] Amin K.M., Rahman D.E.A., Allam H.A., El-Zoheiry H.H. (2021). Design and synthesis of novel coumarin derivatives as potential acetylcholinesterase inhibitors for Alzheimer’s disease. Bioorg. Chem..

[43] Li X., Yu Y., Tu Z. (2021). Pyrazole Scaffold Synthesis, Functionalization, and Applications in Alzheimer’s Disease and Parkinson’s Disease Treatment (2011–2020). Molecules.

[44] Benazzouz-Touami A., Chouh A., Halit S., Terrachet-Bouaziz S., Makhloufi-Chebli M., Ighil-Ahriz K., Silva A.M.S. (2022). New Coumarin-Pyrazole hybrids: Synthesis, Docking studies and Biological evaluation as potential cholinesterase inhibitors. J. Mol. Struct..

[45] Shi D.-H., Min W., Song M., Si X.-X., Li M.-C., Zhang Z., Liu Y.-W., Liu W.-W. (2020). Synthesis, characterization, crystal structure and evaluation of four carbazole-coumarin hybrids as multifunctional agents for the treatment of Alzheimer’s disease. J. Mol. Struct..

[46] Song M.Q., Min W., Wang J., Si X.X., Wang X.J., Liu Y.W., Shi D.H. (2021). Design, synthesis and biological evaluation of new carbazole-coumarin hybrids as dual binding site inhibitors of acetylcholinesterase. J. Mol. Struct..

[47] Tharamak S., Wisarutwanit T., Songoen W., Saparpakorn P., Pluempanupat W. (2023). Synthesis and Acetylcholinesterase Inhibitory Evaluation of Coumarin-Linked Carbazole Derivatives. ChemistrySelect.

[48] Gharat R., Prabhu A., Khambete M.P. (2022). Potential of triazines in Alzheimer’s disease: A versatile privileged scaffold. Arch. Pharm..

[49] Wu W.-L., Wen Z.-Y., Qian J.-J., Zou J.-P., Liu S.-M., Yang S., Qin T., Yang Q., Liu Y.-H., Liu W.-W. (2022). Design, synthesis, characterization and evaluation of 1,3,5-triazine-benzimidazole hybrids as multifunctional acetylcholinesterases inhibitors. J. Mol. Struct..

[50] Zhang X., Wang J., Zou J., Cao Y., Xu X., Ding B., Liu W., Ma S., Shi D. (2024). Design, Synthesis and Anticholinesterase Activity of Coumarin-1,3,5-triazine Derivatives. ChemistrySelect.

[51] Sharma A., Nuthakki V.K., Gairola S., Singh B., Bharate S.B. (2022). A Coumarin−Donepezil Hybrid as a Blood−Brain Barrier Permeable Dual Cholinesterase Inhibitor: Isolation, Synthetic Modifications, and Biological Evaluation of Natural Coumarins. ChemMedChem.

[52] Sharma A., Bharate S.B. (2023). Synthesis and Biological Evaluation of Coumarin Triazoles as Dual Inhibitors of Cholinesterases and β-Secretase. ACS Omega.

[53] Tahir T., Ashfaq M., Saleem M., Rafiq M., Shahzad M.I., Kotwica-Mojzych K., Mojzych M., Scaffolds P. (2021). Phenols and Derivatives of Azo Moiety: Current Therapeutic Perspectives. Molecules.

[54] Sepehri S., Saeedi M., Larijani B., Mahdavi M. (2022). Recent developments in the design and synthesis of benzylpyridinium salts: Mimicking donepezil hydrochloride in the treatment of Alzheimer’s disease. Front. Chem..

[55] Kühnl J., Roggenkamp D., Gehrke S.A., Stäb F., Wenck H., Kolbe L., Neufang G. (2015). Licochalcone A activates Nrf2 in vitro and contributes to licorice extract-induced lowered cutaneous oxidative stress in vivo. Exp. Dermatol..

[56] Liu H., Liu X., Fan H., Tang J., Gao X., Liu W.-K. (2014). Design, synthesis and pharmacological evaluation of chalcone derivatives as acetylcholinesterase inhibitors. Bioorg. Med. Chem..

[57] Lin T.H., Chang K.H., Chiu Y.J., Weng Z.K., Sun Y.C., Lin W., Lee-Chen G.J., Chen C.M. (2022). Neuroprotective Action of Coumarin Derivatives through Activation of TRKB-CREB-BDNF Pathway and Reduction of Caspase Activity in Neuronal Cells Expressing Pro-Aggregated Tau Protein. Int. J. Mol. Sci..

[58] Hasan A.H., Shakya S., Hussain F.H.S., Murugesan S., Chander S., Pratama M.R.F., Jamil S., Das B., Biswas S., Jamalis J. (2023). Design, synthesis, anti-acetylcholinesterase evaluation and molecular modelling studies of novel coumarin-chalcone hybrids. J. Biomol. Struct. Dyn..

[59] Hasan A.H., Abdulrahman F.A., Obaidullah A.J., Alotaibi H.F., Alanazi M.M., Noamaan M.A., Murugesan S., Amran S.I., Bhat A.R., Jamalis J. (2023). Discovery of Novel Coumarin-Schiff Base Hybrids as Potential Acetylcholinesterase Inhibitors: Design, Synthesis, Enzyme Inhibition, and Computational Studies. Pharmaceuticals.

[60] Jana A., Bhattacharjee A., Das S.S., Srivastava A., Choudhury A., Bhattacharjee R., De S., Perveen A., Iqbal D., Gupta P.K. (2022). Molecular Insights into Therapeutic Potentials of Hybrid Compounds Targeting Alzheimer’s Disease. Mol. Neurobiol..

[61] de Sena Murteira Pinheiro P., Franco L.S., Montagnoli T.L., Fraga C.A.M. (2024). Molecular hybridization: A powerful tool for multitarget drug discovery. Expert. Opin. Drug Discov..

[62] Guo J., Mi Z., Jiang X., Zhang C., Guo Z., Li L., Gu J., Zhou T., Bai R., Xie Y. (2021). Design, synthesis and biological evaluation of potential anti-AD hybrids with monoamine oxidase B inhibitory and iron-chelating effects. Bioorg. Chem..

[63] Roberts D., Brunskill S., Doree C., Williams S., Howard J., Hyde C., Roberts D. (2007). Oral deferiprone for iron chelation in people with thalassaemia. Cochrane Database of Systematic Reviews.

[64] Liu W., Wu L., Liu W., Tian L., Chen H., Wu Z., Wang N., Liu X., Qiu J., Feng X. (2022). Design, synthesis and biological evaluation of novel coumarin derivatives as multifunctional ligands for the treatment of Alzheimer’s disease. Eur. J. Med. Chem..

[65] Pourabdi L., Küçükkılınç T.T., Khoshtale F., Ayazgök B., Nadri H., Alashti F.F., Forootanfar H., Akbari T., Shafiei M., Foroumadi A. (2022). Synthesis of New 3-Arylcoumarins Bearing N-Benzyl Triazole Moiety: Dual Lipoxygenase and Butyrylcholinesterase Inhibitors With Anti-Amyloid Aggregation and Neuroprotective Properties Against Alzheimer’s Disease. Front. Chem..

[66] Mphahlele M.J., Agbo E.N., Gildenhuys S., Setshedi I.B. (2019). Exploring Biological Activity of 4-Oxo-4H-furo [2,3-h]chromene Derivatives as Potential Multi-Target-Directed Ligands Inhibiting Cholinesterases, β-Secretase, Cyclooxygenase-2, and Lipoxygenase-5/15. Biomolecules.

[67] Koh S.-H., Kim S.H., Kwon H., Park Y., Kim K.S., Song C.W., Kim J., Kim M.-H., Yu H.-J., Henkel J.S. (2003). Epigallocatechin gallate protects nerve growth factor differentiated PC12 cells from oxidative-radical-stress-induced apoptosis through its effect on phosphoinositide 3-kinase/Akt and glycogen synthase kinase-3. Mol. Brain Res..

[68] Khan S.A., Akhtar M.J., Gogoi U., Meenakshi D.U., Das A. (2023). An Overview of 1,2,3-triazole-Containing Hybrids and Their Potential Anticholinesterase Activities. Pharmaceuticals.

[69] Wang H., Su M., Shi X., Li X., Zhang X., Yang A., Shen R. (2023). Design, Synthesis, Calculation and Biological Activity Studies Based on Privileged Coumarin Derivatives as Multifunctional Anti-AD Lead Compound. Chem. Biodivers..

[70] Di Marzo V. (2006). Endocannabinoids: Synthesis and degradation. Reviews of Physiology, Biochemistry and Pharmacology.

[71] Rampa A., Bartolini M., Bisi A., Belluti F., Gobbi S., Andrisano V., Ligresti A., Di Marzo V. (2021). The first dual ChE/FAAH inhibitors: New perspectives for Alzheimer’s disease?. ACS Med. Chem. Lett..

[72] Montanari S., Allarà M., Scalvini L., Kostrzewa M., Belluti F., Gobbi S., Naldi M., Rivara S., Bartolini M., Ligresti A. (2021). New Coumarin derivatives as cholinergic and cannabinoid system modulators. Molecules.

[73] Montanari S., Scalvini L., Bartolini M., Belluti F., Gobbi S., Andrisano V., Ligresti A., Di Marzo V., Rivara S., Mor M. (2016). Fatty Acid Amide Hydrolase (FAAH), Acetylcholinesterase (AChE), and Butyrylcholinesterase (BuChE): Networked Targets for the Development of Carbamates as Potential Anti-Alzheimer’s Disease Agents. J. Med. Chem..

[74] Canepa E., Parodi-Rullan R., Vazquez-Torres R., Gamallo-Lana B., Guzman-Hernandez R., Lemon N.L., Angiulli F., Debure L., Ilies M.A., Østergaard L. (2023). FDA-approved carbonic anhydrase inhibitors reduce amyloid β pathology and improve cognition, by ameliorating cerebrovascular health and glial fitness. Alzheimers Dement..

[75] Zahedi N.A., Mohammadi-Khanaposhtani M., Rezaei P., Askarzadeh M., Alikhani M., Adib M., Mahdavi M., Larijani B., Niakan S., Tehrani M.B. (2023). Dual functional cholinesterase and carbonic anhydrase inhibitors for the treatment of Alzheimer’s disease: Design, synthesis, in vitro, and in silico evaluations of coumarin-dihydropyridine derivatives. J. Mol. Struct..

[76] Berrino E., Carradori S., Carta F., Melfi F., Gallorini M., Poli G., Tuccinardi T., Fernández-Bolaños J.G., López Ó., Petzer J.P. (2023). A Multitarget Approach against Neuroinflammation: Alkyl Substituted Coumarins as Inhibitors of Enzymes Involved in Neurodegeneration. Antioxidants.

